# T cell-mediated immune function in gastric cancer: a bibliometric overview of the last 20 years (2006-2025)

**DOI:** 10.3389/fonc.2026.1874408

**Published:** 2026-07-13

**Authors:** Tao Wang, Li-Li Li, Chun-Yan Wang, Wen-Jing Sui, Mai-Qing Yang

**Affiliations:** 1 Medical Insurance Affairs Center, Changyi City Healthcare Security Bureau, Changyi, Shandong, China; 2 Department of Pathology, Weifang People’s Hospital (First Affiliated Hospital of Shandong Second Medical University), Weifang, Shandong, China; 3 Department of Family Planning, Weifang People’s Hospital (First Affiliated Hospital of Shandong Second Medical University), Weifang, Shandong, China; 4 School of Clinical Medicine, Weifang People’s Hospital, Shandong Second Medical University, Weifang, Shandong, China

**Keywords:** bibliometric analysis, Citespace, gastric cancer, T cell, VOSviewer

## Abstract

**Objective:**

Gastric cancer (GC) causes massive cancer-related deaths worldwide. T cell-mediated immunity plays a vital role in anti-tumor responses, immune surveillance and immunotherapy efficacy for GC. Although numerous relevant studies have been published, no standardized multi-database bibliometric analysis has illustrated the overall development trajectory, cooperation patterns and research theme evolution of this field from 2006 to 2025. This bibliometric study aimed to quantify publication trends, collaborative networks and research hotspots of GC-related T cell immunity over the past two decades.

**Methods:**

Literature restricted to reviews with T cell immune regulation in GC published from 2006 to 2025 was retrieved from the Web of Science Core Collection (WoSCC), PubMed, Scopus. CiteSpace, VOSviewer were applied for bibliometric and visual analysis, encompassing publication trends, core authors, institutions, countries, journals, keywords, and research hotspots.

**Results:**

In total, 2,476 eligible publications were enrolled. Annual publications trended upward and surged after 2017, reaching 337 papers in 2025. Collaboration analysis showed China led global publication and citation outputs across the 20 years, while high bibliometric metrics only reflect regional research scale rather than superior research quality or clinical translation capacity. Fudan University, Frontiers in Immunology, Suk Ki Tae and Giovanni Targher were the most productive institution, journal, author and most co-cited author, respectively. Keywords including immune infiltration, tumor immune microenvironment, drug resistance and chemotherapy have gained rising popularity recently. Dynamic keyword shifts were closely linked to emerging single-cell sequencing technology and immune checkpoint inhibitor clinical trials. Importantly, keyword co-occurrence and clustering only show literature thematic patterns, not direct biological mechanisms or causal tumor immunology relationships.

**Discussion:**

This bibliometric study systematically and objectively summarizes the research status of T cells in GC, offering valuable insights and guidance for subsequent studies.

**Conclusions:**

GC-related T cell immunity research has grown rapidly from 2006 to 2025, focusing mainly on tumor microenvironment regulation and immunotherapy. This work clarifies the field’s research status and evolutionary trends, guiding future relevant investigations. Unlike previous single-database bibliometric analyses, this study adopts three mainstream databases, standardized data processing procedures, and providing novel supplementary insights for current evidence.

## 1 Introduction

Globally, Gastric cancer (GC) remains one of the most frequently diagnosed malignancies and a leading cause of cancer-related mortality (1–3). Its incidence exhibits marked geographical and gender differences, with substantially higher rates in East Asia (China, Japan, South Korea) than in Europe and North America, and a male-to-female ratio of approximately 2:1 (2, 4–6). Adenocarcinoma constitutes the predominant histological subtype, followed by mucinous adenocarcinoma, signet ring cell carcinoma, and undifferentiated carcinoma (5, 7–9). Owing to the paucity of specific early-stage symptoms, the majority of GC patients are diagnosed at advanced stages, characterized by rapid disease progression, high rates of lymph node and distant metastasis, and consequently poor prognosis with low 5-year survival rates (3, 9–11).

The immune system exerts a critical influence on the initiation, progression, and metastatic dissemination of GC, with T cell-mediated immune function serving as the central component of the anti-tumor immune response (12–14). T cells, especially cytotoxic T lymphocytes (CD8+ T cells), can specifically recognize and kill tumor cells, while helper T cells (CD4+ T cells) regulate the immune response by secreting cytokines, and regulatory T cells (Tregs) inhibit the anti-tumor immune response, leading to immune escape of tumor cells (15–19). The dysfunction or abnormal regulation of T cells directly affects the progression of GC and the efficacy of immunotherapy. In recent years, with the in-depth research on tumor immunology, immunotherapies such as immune checkpoint inhibitors (ICIs), CAR-T cell therapy, and neoantigen vaccines have brought new hope for the treatment of advanced GC, and the regulatory mechanisms of T cell-mediated immune function have become a research hotspot in the field of GC immunology (13, 20–22).

Bibliometric analysis is a quantitative research method that uses mathematical and statistical methods to analyze the distribution, characteristics, and trends of literature in a specific field, which can intuitively reflect the research status, hotspots, and development trends of the field. It has been widely used in medical research to sort out research progress and provide reference for future research (23–25). Over the past 20 years, a large number of literature on T cell-mediated immune function in GC have been published, but existing bibliometric reports on GC tumor immunity mostly rely on single database data, lack unified standardized data screening workflows, and rarely combine the evolution of research hotspots with clinical and technological breakthroughs. Therefore, this study conducted a bibliometric analysis of literature related to T cell-mediated immune function in GC published from 2006 to 2025 based on three authoritative databases with standardized retrieval, deduplication and analysis parameters, aiming to clarify the research hotspots, core teams, and development trends, provide a comprehensive reference for researchers in this field, and promote the in-depth development of related research. We emphasize in advance that all quantitative metrics (publication volume, citation counts, keyword frequency) in this work only describe the distribution of published papers, and cannot be used to rank the overall scientific quality or clinical impact of institutions, authors or research directions.

## 2 Materials and methods

### 2.1 Data collection

A systematic search was performed in three authoritative databases: PubMed, Web of Science Core Collection (WoSCC), and Scopus, using a combination of controlled vocabulary and free-text terms to identify studies taking T cell immune regulation as the primary research focus on T cell and GC (26). Publications related to T cells in gastric cancer from January 1, 2006, to December 31, 2025, were retrieved. Only English journal articles and reviews focusing on human subjects were included to ensure quality and relevance. The search was conducted on April 10, 2026. The search terms were presented as follows: PubMed: (Gastric neoplasms[MeSH Terms] OR (gastric cancer OR stomach cancer OR gastric carcinoma OR gastric neoplasms OR GC)[Title/Abstract]) AND ((“T cell” OR “T cells” OR “T lymphocyte” OR “T lymphocytes” OR CD4+ OR CD8+ OR “cytotoxic T lymphocyte” OR CTL OR “regulatory T cell” OR Treg)[Title/Abstract]) AND “2006/01/01”[Date - Publication]: “2025/12/31” AND Humans[MeSH Terms] AND English[language] AND (Journal Article[pt] OR Review[pt]); WoSCC: TS=((gastric cancer OR stomach cancer OR gastric carcinoma OR gastric neoplasms OR GC) AND (“T cell” OR “T cells” OR “T lymphocyte” OR “T lymphocytes” OR CD4+ OR CD8+ OR “cytotoxic T lymphocyte” OR CTL OR “regulatory T cell” OR Treg)) AND PY = 2006–2025 AND DT=(Article OR Review) AND LANGUAGE=English. Scopus: TITLE-ABS-KEY ((gastric cancer OR stomach cancer OR gastric carcinoma OR gastric neoplasms OR GC) AND (“T cell” OR “T cells” OR “T lymphocyte” OR “T lymphocytes” OR CD4+ OR CD8+ OR “cytotoxic T lymphocyte” OR CTL OR “regulatory T cell” OR Treg)) AND PUBYEAR>2005 AND PUBYEAR<2026 AND DOCTYPE (Article OR Review) AND LANGUAGE (English).

Detailed reproducible data processing procedures: (1) Initial retrieval: A total of 18,960 records were initially identified (8,867 from WoSCC, 4,212 from Scopus, and 5881 from PubMed). (2) Cross-database deduplication: All literature metadata (title, DOI, authors, journal, publication year) were imported into EndNote 20 for primary automatic duplicate removal; remaining potential duplicate entries with inconsistent metadata were manually screened by two researchers according to DOI and full-text title matching, removing 9,925 duplicate records. (3) Language filtering: Excluded 45 non-English articles, retaining 9,035 records. (4) Document type screening: Excluded conference abstracts, meeting papers, letters, retracted articles, leaving 5,915 articles for full-text evaluation.

Full-text inclusion standard: Two investigators independently screened titles, abstracts, and full texts; papers only briefly mentioning T cell markers without exploring T cell immune function as core research content were excluded, eliminating 3,439 irrelevant studies. Finally, 2,476 eligible studies were included for bibliometric analysis.

Standardization of metadata: First, VOSviewer’s built-in thesaurus function was used to automatically merge common variants. Second, all extracted metadata (author names, institutional affiliations, journal names) were manually cross-checked to correct for inconsistencies (e.g., “Fudan Univ” vs. “Fudan University”). For authors with multiple abbreviated spellings, unified full names were adopted; Chinese institutions with different translation versions were corrected to official English names; journal abbreviations were converted to full official journal titles to eliminate analysis bias caused by inconsistent naming. VOSviewer and CiteSpace were retrieved and imported for bibliometric analysis (27, 28).

### 2.2 Data analysis and visualization

Two researchers independently extracted the data from the retrieved literature, including the title, author, affiliation, country, publication year, journal, keywords, citations, and abstract. Disagreements between the two researchers were resolved through discussion or consultation with a third researcher. The extracted data were sorted and cleaned using Excel, including removing duplicate data, correcting typos, and standardizing the names of institutions and countries. The software tools used for bibliometric analysis were VOSviewer (version 1.6.20, Leiden University, The Netherlands) and CiteSpace (version 6.3. R1; Drexel University, PA, USA). Complete software parameter settings are listed below to ensure reproducibility: (1) VOSviewer parameters: Co-occurrence threshold of keywords = 30; co-authorship counting method = full counting; cluster resolution parameter = 1.0; network pruning adopted automatic attractor pruning. (2) CiteSpace parameters: Time slice = 2 year (2006–2025); top N = top 100 most cited items per slice; Pruning: Pathfinder network scaling with a pruning strategy to retain the main skeleton of the network. These parameters were chosen based on standard practices in the field to avoid over-fragmentation of clusters.

**Figure 1:**
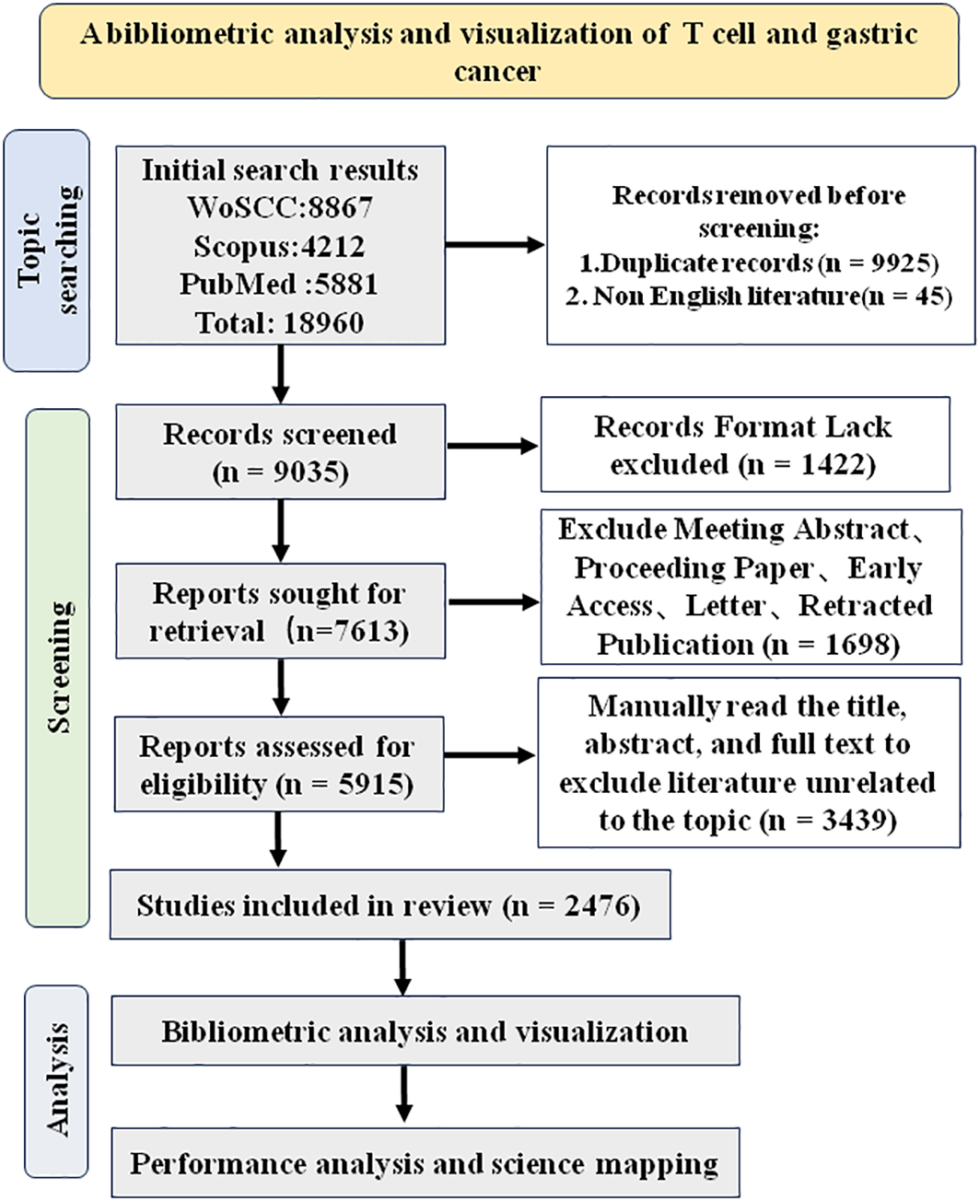
Study flow chart.

The specific analysis contents included: (1) Publication trend analysis: Statistical analysis of the number of annual publications and citations to clarify the development trend of the field over the past 20 years. We further correlated annual output changes with landmark events such as the launch of PD-1/PD-L1 clinical trials and single-cell immune sequencing technology to interpret trend drivers. (2) Core author and cooperation network analysis: Identify core authors based on the number of publications and citations, and analyze the cooperation relationship between authors. Author output and citation counts only reflect literature output volume and citation popularity within this dataset, and cannot directly represent the scientific innovation or clinical transformation value of individual research. (3) Institutional and national cooperation analysis: Analyze the distribution of research institutions and countries, and the cooperation network between institutions and countries. (4) Journal analysis: Analyze the distribution of literature in journals, including the number of publications and journal category. (5) Keyword analysis: Analyze the co-occurrence, clustering, and evolution of keywords to identify research hotspots and development trends. Keyword co-occurrence clustering only reflects the aggregation law of literature topics, and cannot be directly regarded as causal evidence of biological immune mechanisms. (6) Citation analysis: Analyze the top cited literature and their influence in the field. In this study, variables are expressed as numbers and percentages. No comparisons were made; therefore, no P value was set. The search tactics are shown in **Figure 1**.

## 3 Results

### 3.1 Analysis of publication quantity and trends

**Figure 2:**
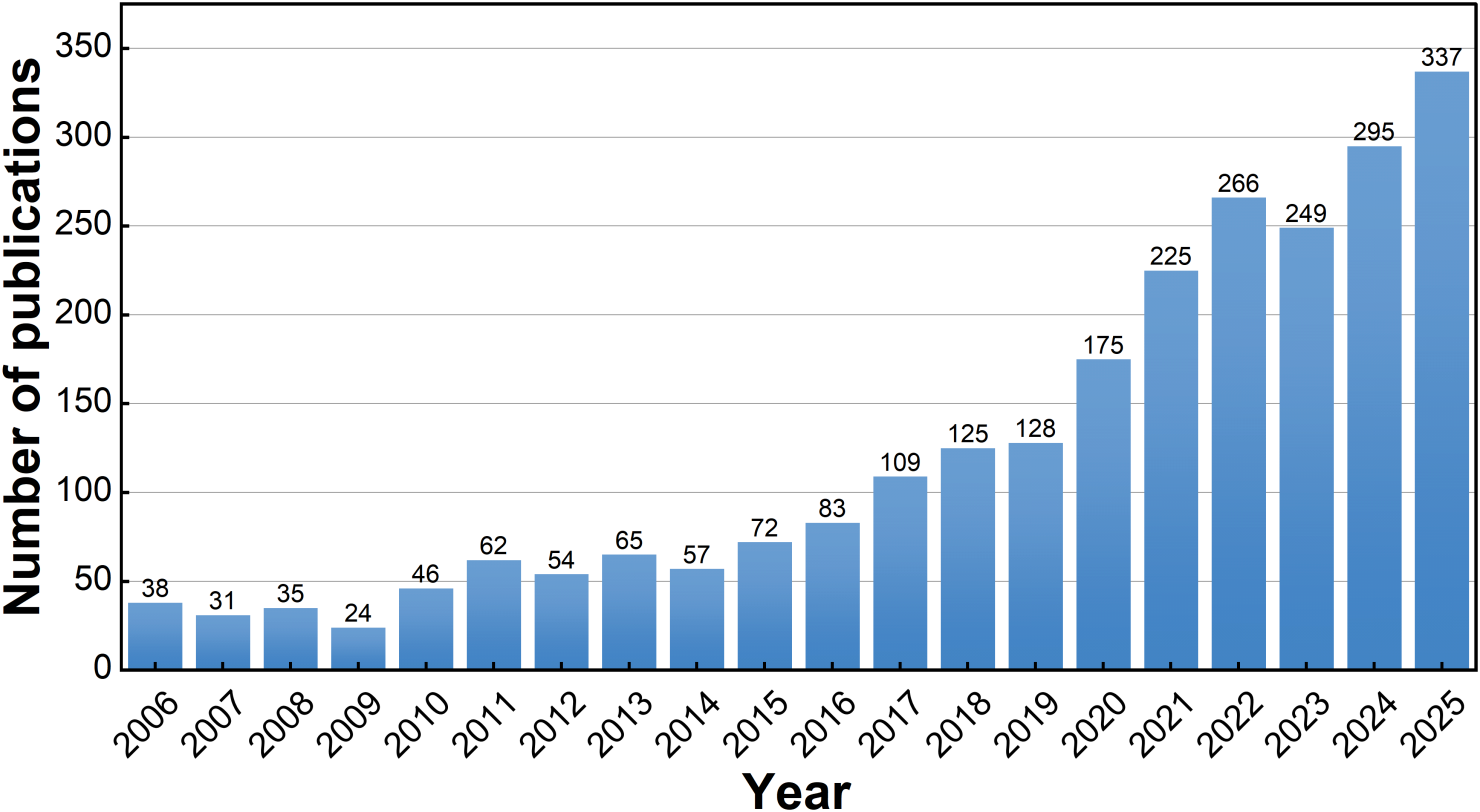
Annual number of publications related to T cell and gastric cancer.

A total of 2,476 related research documents related to the involvement of T cell in GC were included in the analysis after selecting. The temporal analysis of publications demonstrates a clear long-term growth trajectory in the field. After a period of low and fluctuating productivity between 2006 and 2013 (peaking at 65 documents in 2013), the field entered a phase of general growth from 2014 onward. A notable acceleration is observed from 2020, with publication numbers growing substantially between 2020 (175 documents) and 2022 (266 documents), and reaching an all-time high of 337 publications in 2025. This growth trend coincides with the large-scale clinical application of ICIs after 2017 and the popularization of single-cell sequencing technology after 2019, which jointly promoted the surge of related research output. This pattern confirms a significant and growing global research interest in the subject, with the pace of research output increasing dramatically in recent years (**Figure 2**).

### 3.2 Distribution of countries and institutions

#### 3.2.1 Contributions of countries

**Figure 3:**
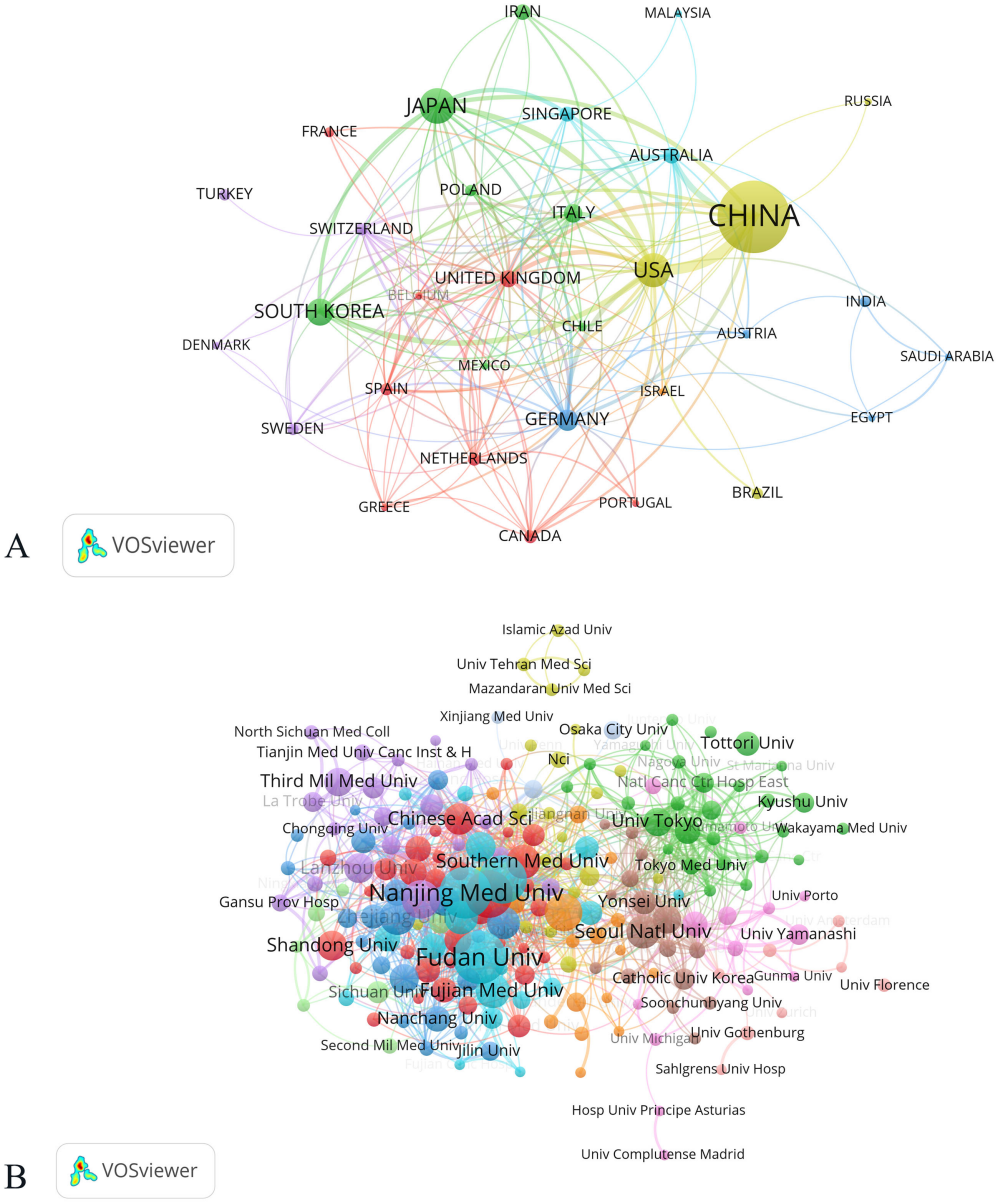
Visualization of countries and institutions. **(A)** International cooperation in related research occurred between different countries. **(B)** Cooperation in related research occurred between different institutions.

This bibliometric analysis reveals distinct patterns in research productivity and collaboration within the field. China records the largest cumulative paper count of 1,636 documents and 44,921 citations among all nations, a figure substantially higher than that of other leading nations such as Japan and the United States of America (USA) (**Table 1**). It should be emphasized that higher publication and citation volume only reflects the scale of regional research output, and does not equal overall superiority in research quality or clinical translational achievements. Despite its high volume, China maintains a central position in the global collaboration network, as evidenced by the VOSviewer visualization, which identifies it as the key hub connecting major research economies including the USA, Japan, Australia, and South Korea (**Figure 3A**). The data further highlights a clear regional clustering of collaboration: East Asian countries form a tightly knit sub-network, while Europe and North America represent another major cluster. Collectively, these findings confirm China’s dual role as both the primary contributor to global research output and a critical bridge for international scientific cooperation.

**Table 1:** Top 15 high-output countries/regions in the field.

Id	Country	Documents	Citations
1	CHINA	1,636	44,921
2	JAPAN	285	15,255
3	USA	254	15,031
4	SOUTH KOREA	140	7,654
5	GERMANY	82	3,685
6	ITALY	57	2,659
7	UNITED KINGDOM	47	2,715
8	AUSTRALIA	41	2,602
9	IRAN	37	836
10	SINGAPORE	33	2,377
11	NETHERLANDS	22	1,414
12	CANADA	22	788
13	SPAIN	19	1,458
14	SWITZERLAND	19	1,149
15	TURKEY	18	240

#### 3.2.2 Contributions of institutions

The analysis of institutional productivity and collaboration reveals a clear concentration of research activity in Chinese academic institutions. Among the top 15 high-output organizations, 14 are based in China, with Fudan University (116 documents) and Nanjing Medical University (110 documents) leading in publication volume, underscoring their central role in advancing the field. Notably, Seoul National University (South Korea) is the only non-Chinese institution in the ranking; while its document count (46) is lower than many Chinese peers, it records the highest citation count (4,942), indicating substantial global academic influence (**Table 2**). The VOSviewer collaboration network further illustrates this pattern: leading Chinese institutions, particularly Fudan University and Nanjing Medical University, are positioned as key hubs, maintaining extensive collaborative ties with both domestic partners and international institutions across Asia, Europe, and North America (**Figure 3B**). This confirms that Chinese universities not only drive research output but also play a critical role in shaping the field’s collaborative landscape.

**Table 2:** Top 15 high-output institutions in the field.

Id	Organization	Documents	Citations
1	Fudan Univ	116	3,000
2	Nanjing Med Univ	110	2,653
3	Shanghai Jiao Tong Univ	98	3,188
4	Sun Yat Sen Univ	94	3,708
5	Zhengzhou Univ	61	2,063
6	Soochow Univ	58	1,859
7	Zhejiang Univ	53	1,675
8	Southern Med Univ	48	2,497
9	Fujian Med Univ	48	908
10	Seoul Natl Univ	46	4,942
11	Nanjing Univ	46	1,285
12	China Med Univ	44	3,099
13	Chinese Acad Sci	41	869
14	Jiangsu Univ	38	1,347
15	Lanzhou Univ	38	654

### 3.3 Analysis of authors

**Figure 4:**
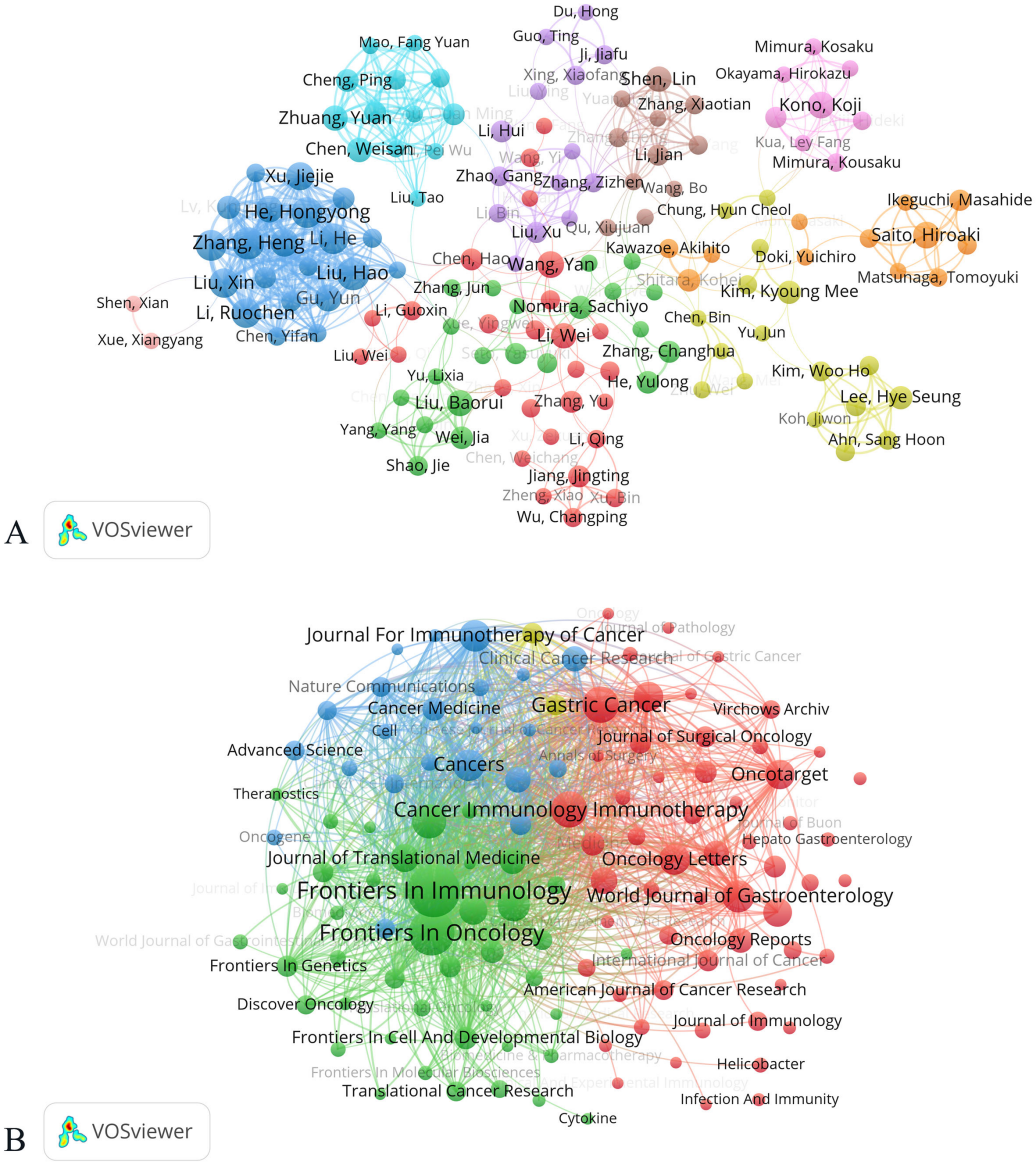
Visualization of authors and journals. **(A)** Cooperation in related research occurred between different authors. **(B)** Cooperation in related research occurred between different journals.

The analysis of high-output authors and their collaboration networks highlights a diverse distribution of productivity and impact. Suk, Ki Tae is the most productive author with 14 publications, while Targher, Giovanni stands out for his exceptional citation impact (2,023 citations), far exceeding peers with similar publication counts (**Table 3**). After manual disambiguation of author names, repeated citation records caused by inconsistent name abbreviations were eliminated, ensuring the reliability of author citation statistics. The VOSviewer map further illustrates clear, geographically defined author clusters: large, interconnected networks of Chinese researchers form the largest collaborative groups, while European and East Asian authors form their own distinct clusters (**Figure 4A**). This structure indicates that research collaboration is largely organized around regional and institutional ties, with limited cross-cluster integration between different geographic author communities. We note that the ranking of authors by publication or citation metrics does not equate to their standing as leading clinical or basic science experts in GC T cell immunology across the whole field.

**Table 3:** Top 15 high-output authors in the field.

Id	Author	Documents	Citations
1	Suk, Ki Tae	14	340
2	Nobili, Valerio	13	1,258
3	Abenavoli, Ludovico	13	603
4	Roomba, Rohit	12	1,302
5	Byrne, Christopher D.	12	1,245
6	Gupta, Hari Priya	12	217
7	Targher, Giovanni	11	2,023
8	Schnabl, Bernd	11	1,308
9	Alisi, Anna	11	980
10	Bergheim, Ina	10	456
11	Kim, Dong Joon	10	181
12	Tilg, Herbert	9	1,403
13	Nieuwdorp, Max	9	703
14	Hekmatdoost, Azita	9	552
15	Ganesan, Raja	9	54

### 3.4 Analysis of journals

The analysis of high-output journals and their co-citation networks identifies key publication venues and thematic divisions in the field. *Frontiers in Immunology* leads in publication volume (94 documents), followed by *Frontiers in Oncology* (71 documents), indicating their role as primary outlets for research in this domain. Journals like *Gastric Cancer* and *Cancer Immunology Immunotherapy* also feature prominently, balancing high output with strong citation impact (**Table 4**). The VOSviewer visualization further illustrates three distinct journal clusters: a green cluster centered on immunology and translational medicine, a blue cluster covering general oncology and cancer research, and a red cluster focused on gastric cancer and clinical oncology (**Figure 4B**). This structure reflects the field’s core thematic areas, with clear boundaries between basic immunology, general oncology, and GC-specific research.

**Table 4:** Top 15 high-output journals in the field.

Id	Source	Documents	Citations
1	Frontiers In Immunology	94	2,216
2	Frontiers In Oncology	71	1,198
3	Gastric Cancer	45	2,063
4	Cancer Immunology Immunotherapy	45	1,855
5	Scientific Reports	40	759
6	World Journal of Gastroenterology	36	1,429
7	International Immunopharmacology	35	710
8	Journal For Immunotherapy of Cancer	34	1,056
9	Cancers	34	481
10	Oncology Letters	32	471
11	Oncoimmunology	30	1,434
12	Oncotarget	30	1,432
13	Plos One	29	1,329
14	Journal of Translational Medicine	27	701
15	Bmc Cancer	27	410

### 3.5 Key topics of research hotspots

Keyword analysis was performed to identify research hotspots and evolutionary trends in T cell-mediated immune function in GC from 2006 to 2025.

#### 3.5.1 Analysis of clusters and co-occurrence of keywords

**Figure 5:**
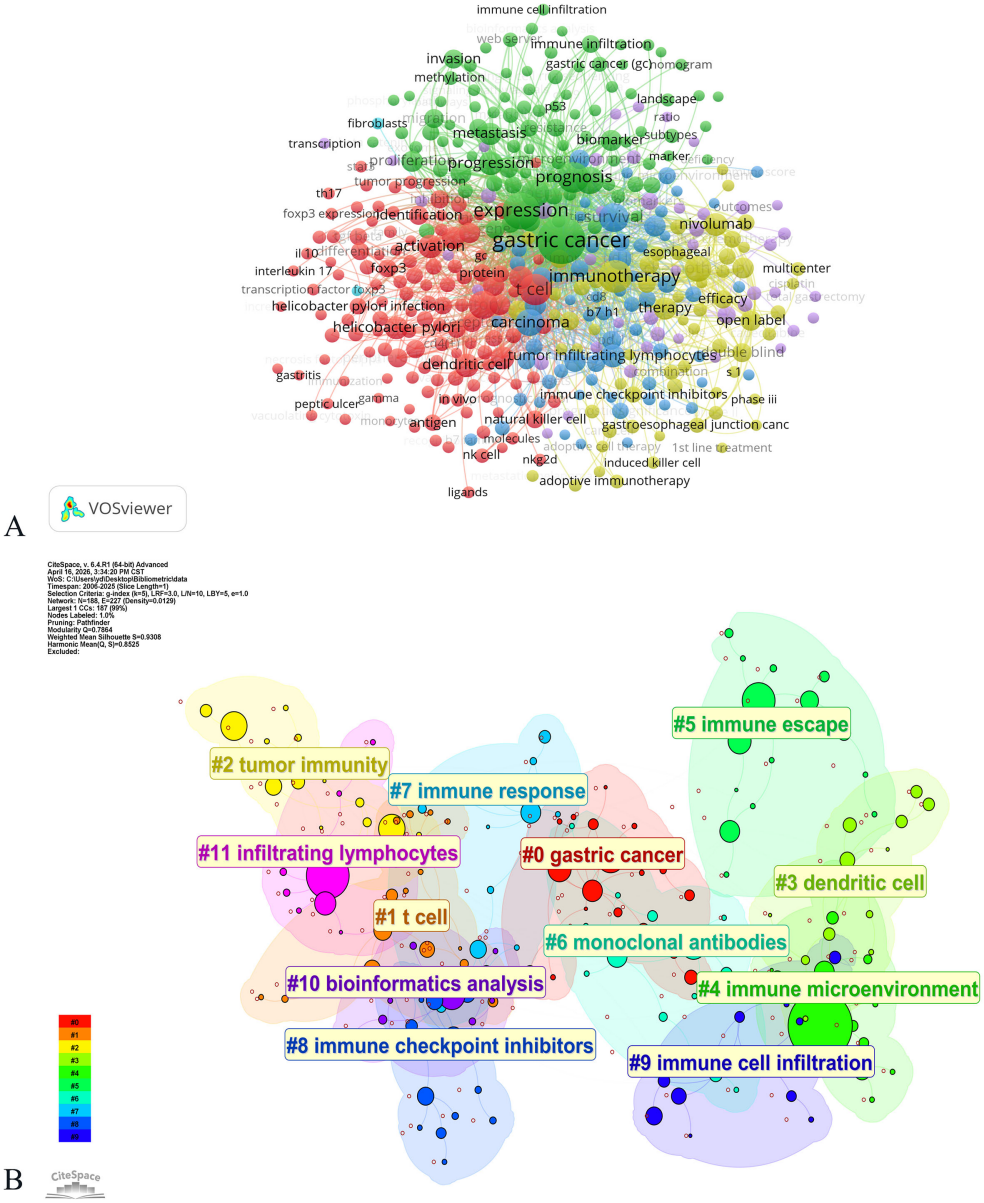
Visualization of keywords.**(A)** Network visualization map of co-citations of keywords. **(B)** Analysis results of hotspot and topic in the field of T cell in gastric cancer.

Keyword co−occurrence networks were constructed using VOSviewer and CiteSpace. High−frequency keywords (frequency ≥ 30) were clustered into eleven major themes (**Figure 5**). The most prominent clusters centered on gastric cancer, T cell, tumor immunity, immune microenvironment, immune escape, immune checkpoint inhibitors, and bioinformatics analysis. The top high−frequency keywords were gastric cancer (1,511 occurrences), expression (637), immunotherapy (453), T cell (387), and prognosis (368), highlighting the core focus on immune mechanisms, prognosis, and immunotherapy development (**Table 5**). We reiterate that these co-occurrence clusters only reflect topical grouping of published papers and cannot independently validate causal biological relationships between T cell phenotypes and GC progression.

**Table 5:** Top 20 keywords with high frequency related to research.

Id	Keyword	Occurrences	Total link strength
1	Gastric Cancer	1,511	9,621
2	Expression	637	4,336
3	Immunotherapy	453	3,149
4	T Cell	387	2,815
5	Prognosis	368	2,575
6	Survival	285	2,129
7	Regulatory T Cell	273	2,174
8	Cell	249	1,453
9	Tumor Microenvironment	245	1,723
10	Carcinoma	230	1,752
11	Chemotherapy	218	1,588
12	Activation	157	1,122
13	Progression	148	1,109
14	Dendritic Cell	145	1,142
15	Helicobacter Pylori	141	987
16	Nivolumab	139	1,061
17	Lymphocytes	138	1,103
18	Tumor Infiltrating Lymphocytes	136	1,142
19	Cancer	134	871
20	Inflammation	129	929

#### 3.5.2 Burst detection and overlay visualization of keywords

**Figure 6:**
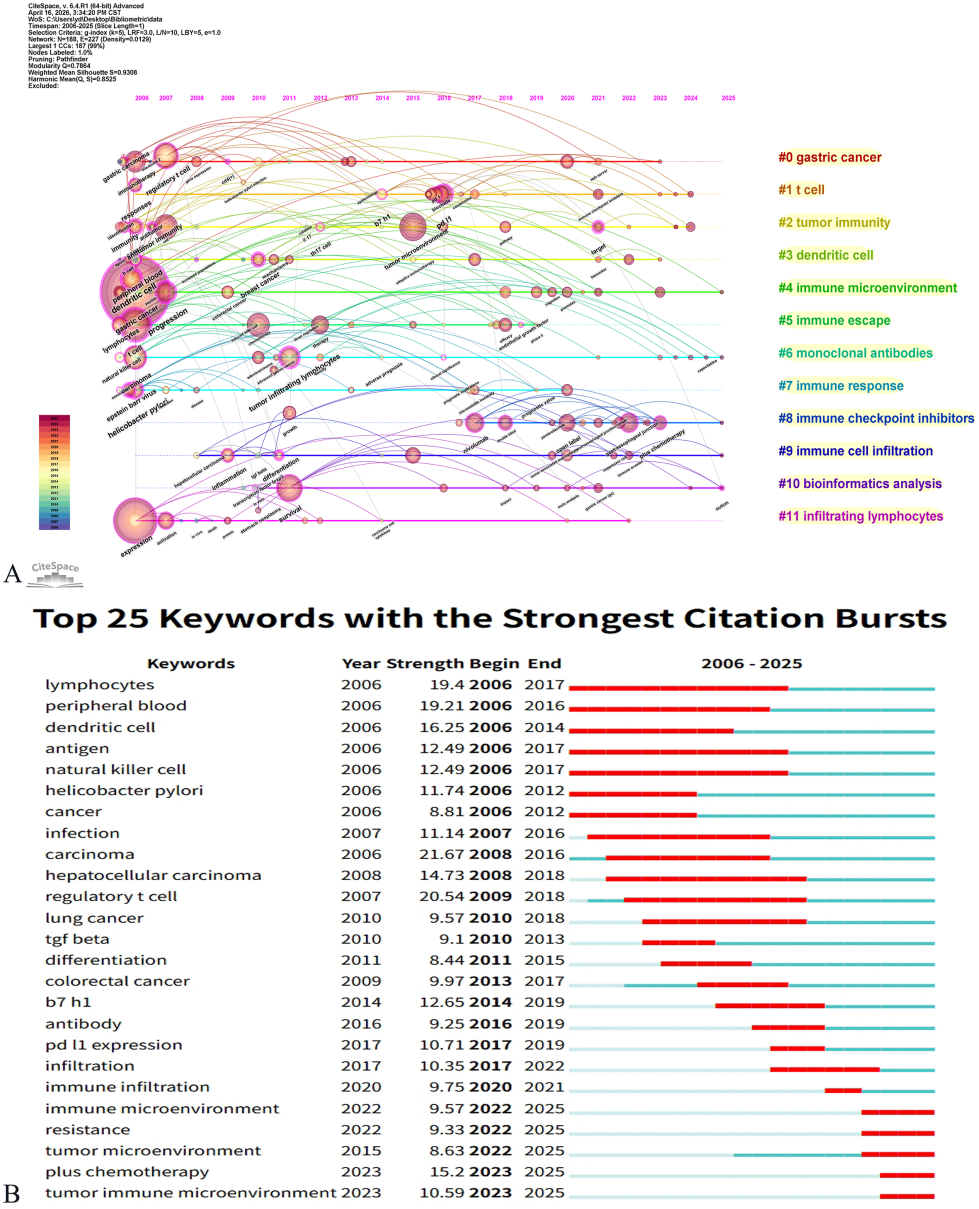
Keywords clustering timeline graph and keywords citation bursts. **(A)** Keywords clustering timeline graph. **(B)** Top 25 keywords with the strongest citation bursts.

Keyword burst detection revealed dynamic evolutionary trends. Early bursts (2006–2015) included lymphocytes, peripheral blood, dendritic cell, and helicobacter pylori, reflecting basic immunological and carcinogenic mechanism studies. Mid−period bursts (2010–2020) were dominated by regulatory T cell, PD-L1 expression, and B7-H1, corresponding to the rise of immune checkpoint research. Recent sustained bursts (2020–2025) included immune microenvironment, tumor microenvironment, and plus chemotherapy, indicating that the tumor immune microenvironment and combination immunotherapy have become the leading frontiers (**Figure 6**). This hotspot shift is closely linked to the maturity of single-cell transcriptome technology and multiple phase III clinical trials of combined immunochemotherapy for advanced GC published after 2020.

In summary, research in T cell-mediated immune function in GC has shifted from basic immune mechanisms to clinical translational applications, with the tumor immune microenvironment and combination therapy as the key future directions.

## 4 Discussions

### 4.1 Summary of findings

This study conducted a comprehensive bibliometric analysis of 2,476 research documents related to the involvement of T cells in GC from 2006 to 2025, systematically summarizing the research status, distribution characteristics, and development trends of this field. The temporal trend analysis showed that the research output in this field has experienced two distinct stages: a low and fluctuating stage from 2006 to 2013, followed by a general growth stage from 2014 onward, with a significant acceleration since 2020 and an all-time high of 337 publications in 2025, reflecting the growing global attention to T cell-related research in GC. In terms of regional and institutional distribution, China dominates the global research output with 1,636 documents and 44,921 citations, serving as a core hub in the global collaborative network; among the top 15 high-output institutions, 14 are Chinese, with Fudan University and Nanjing Medical University leading the way, while Seoul National University (South Korea) stands out for its high citation impact. In terms of authors, Suk, Ki Tae is the most productive author, and Targher, Giovanni has the highest citation impact, with author collaborations showing obvious regional clustering characteristics. In terms of journals, Frontiers in Immunology and Frontiers in Oncology are the main publication venues, and the journal clusters are divided into three themes: immunology and translational medicine, general oncology, and GC-specific research. Keyword analysis identified 11 major research themes, with the core focus on immune mechanisms, prognosis, and immunotherapy, and the research hotspots have shown a dynamic evolution from basic immunology to clinical translation. Throughout this summary, we distinguish clearly between observable patterns in published literature output and substantive scientific breakthroughs or clinical achievements in the field.

### 4.2 Research hotspots and frontier exploration in T cell of GC field

Based on keyword co-occurrence, clustering, and burst detection, the research hotspots and frontiers of T cell-related research in GC have shown clear evolutionary rules over the past 20 years. The core hotspots of the field are concentrated in seven major directions: gastric cancer, T cell, tumor immunity, immune microenvironment, immune escape, immune checkpoint inhibitors, and bioinformatics analysis, among which gastric cancer, expression, immunotherapy, T cell, and prognosis are the most high-frequency keywords, indicating that the field has always focused on the core issues of T cell-mediated immune response mechanisms in GC, the expression characteristics of related molecules, and the development of immunotherapeutic strategies.

From the perspective of frontier exploration, the research hotspots have undergone three stages of evolution, and we further supplement the technological and clinical driving factors behind each stage’s transformation: (1) Early stage (2006–2015): Focused on basic immunological mechanisms and carcinogenic factors, with burst keywords such as lymphocytes, peripheral blood, dendritic cells, and Helicobacter pylori. Limited by low-throughput immune detection technology at that time, research mainly explored the correlation between peripheral immune cells and GC tumorigenesis. (2) Middle stage (2010–2020): Witnessed the rise of immune checkpoint research, with burst keywords including regulatory T cells, PD-L1 expression, and B7-H1. Driven by the approval of anti-PD-1/PD-L1 ICIs for solid tumors, scholars began to focus on tumor immune escape targets represented by PD-L1. (3) Recent stage (2020–2025): Has formed new frontiers centered on the immune microenvironment, tumor microenvironment, and combination chemotherapy. The popularization of single-cell sequencing technology and positive results of multiple combined immunochemotherapy clinical trials for advanced GC jointly pushed tumor microenvironment regulation and combinatorial therapy to become the most cutting-edge direction in the field.

### 4.3 Relationship between T cell and GC

The bibliometric results collectively delineate the close and multifaceted relationship between T cells and GC, spanning the entire course of GC initiation, progression, prognosis, and therapeutic intervention (12, 15, 21, 29–31). As central components of the adaptive immune system, T cells exert a dual role in GC. on the one hand, they participate in the anti-tumor immune response by recognizing and killing GC cells, and their infiltration level and functional status are closely related to the prognosis of GC patients; on the other hand, abnormal activation or functional exhaustion of T cells (such as regulatory T cells and exhausted T cells) can lead to immune escape of GC cells, promoting tumor progression and metastasis (13, 18, 19, 21).

Keyword co-occurrence analysis further corroborates this relationship. The elevated frequency of terms such as “immune escape” and “immune checkpoint inhibitors” indicates that T cell functional dysregulation constitutes a significant mechanism underlying GC progression (32, 33), while the high attention to “immunotherapy” reflects that targeting T cell function to restore anti-tumor immunity has become an important therapeutic strategy for GC (22). In addition, the close correlation between T cells and the tumor immune microenvironment is also a key part of their relationship with GC-T cells interact with other immune cells (such as dendritic cells, B cells) and stromal cells in the tumor microenvironment, jointly regulating the anti-tumor immune response and affecting the efficacy of immunotherapy (34, 35). Regional and institutional collaboration network analyses reveal that research on the T cell-GC relationship has formed a global collaborative framework, with China occupying a central position in advancing investigation into this association.

### 4.4 Future development

Based on the identified research trends and frontiers, future investigations in T cell-related GC research are anticipated to focus on the following areas: in-depth exploration of the tumor immune microenvironment’s regulatory effect on T cell function for new targets; advancement of combined immunotherapy strategies to improve patient outcomes; wider application of bioinformatics in biomarker screening; and strengthened international and inter-institutional collaboration to accelerate research translation.

## 5 Limitations

This study has several limitations: (1) The included documents are limited to English journal articles and reviews retrieved from three mainstream databases, potentially missing gray literature, non-English high-quality works, conference papers with complete research data, which may produce certain language and database selection bias. (2) This study relies purely on quantitative bibliometric indicators (publication volume, citations, co-occurrence frequency) for analysis, without systematic qualitative evaluation of research innovation, clinical conversion value or experimental design rigor of each included paper. All rankings and trend descriptions derived from these metrics cannot be used to judge the actual scientific quality or clinical significance of individual studies or research teams. (3) VOSviewer-based collaboration analysis only reflects surface co-authorship ties between scholars/institutions, and cannot quantify the depth and academic output of actual cooperation projects. (4) Keyword trend analysis may be affected by database update speed and inconsistent keyword marking standards of different journals, so the prediction of future research trends has certain uncertainty and can only be used as a reference.

## 6 Conclusions

This bibliometric analysis summarizes T cell-related GC research from 2006 to 2025, showing rapid development with China as a core contributor. Over the past 20 years (2006–2025), the research on T cell-mediated immune function in GC has achieved remarkable progress, showing a steady upward development trend. Research hotspots have shifted from basic immunology to clinical translation, focusing on the tumor immune microenvironment and combined immunotherapy. Different from previous single-database bibliometric studies on GC immunity, this work integrates three mainstream retrieval platforms, forms a fully standardized and reproducible literature screening and metadata unification process, and systematically explains the technical and clinical drivers of research hotspot evolution, provides unique supplementary value for the field. Despite limitations, this study provides a comprehensive overview to guide future research, which is expected to bring new breakthroughs for GC diagnosis and treatment.

## Data Availability

The original contributions presented in the study are included in the article/supplementary material. Further inquiries can be directed to the corresponding author.
